# The Virtual Kitchen Challenge—Version 2: Validation of a Digital Assessment of Everyday Function in Older Adults

**DOI:** 10.2196/82092

**Published:** 2026-01-07

**Authors:** Marina Kaplan, Moira McKniff, Stephanie M Simone, Molly B Tassoni, Katherine Hackett, Sophia Holmqvist, Rachel E Mis, Kimberly Halberstadter, Riya Chaturvedi, Melissa Rosahl, Giuliana Vallecorsa, Mijiail D Serruya, Deborah A G Drabick, Takehiko Yamaguchi, Tania Giovannetti

**Affiliations:** 1 Department of Psychology and Neuroscience Temple University Philadelphia, PA United States; 2 Department of Psychiatry Neuropsychology Section Michigan Medicine Ann Arbor, MI United States; 3 Division of General Internal Medicine Icahn School of Medicine at Mount Sinai New York, NY United States; 4 Department of Neurology Dell Medical School University of Texas at Austin Austin, TX United States; 5 Department of Psychology LaSalle University Philadelphia, PA United States; 6 Raphael Center for Neruorestoration Thomas Jefferson University Philadelphia, PA United States; 7 Department of Applied Information Engineering Suwa University of Science Nagano Japan

**Keywords:** everyday function, activities of daily living, assessment, dementia, Alzheimer disease, neuropsychology, cognition, mild cognitive impairment, virtual reality, digital assessment

## Abstract

**Background:**

Conventional methods of functional assessment include subjective self- or informant report, which may be biased by personal characteristics, cognitive abilities, and lack of standardization (eg, influence of idiosyncratic task demands). Traditional performance-based assessments offer some advantages over self- or informant reports but are time-consuming to administer and score.

**Objective:**

This study aims to evaluate the validity and reliability of the Virtual Kitchen Challenge—Version 2 (VKC-2), an objective, standardized, and highly efficient alternative to current functional assessments for older adults across the spectrum of cognitive aging, from preclinical to mild dementia.

**Methods:**

A total of 236 community-dwelling, diverse older adults completed a comprehensive neuropsychological evaluation to classify cognitive status as healthy, mild cognitive impairment, or mild dementia, after adjustment for demographic variables (age, education, sex, and estimated IQ). Participants completed 2 everyday tasks (breakfast and lunch) in a virtual kitchen (VKC-2) using a touchscreen interface to select objects and sequence steps. Automated scoring reflected completion time and performance efficiency (eg, number of screen interactions, percentage of time spent off-screen, interactions with distractor objects). Participants also completed the VKC-2 tasks using real objects (Real Kitchen). All participants and informants for 219 participants completed questionnaires regarding everyday function. A subsample of participants (n=143) performed the VKC-2 again in a second session, 4-6 weeks after the baseline, for retest analyses. Analyses evaluated construct and convergent validity, as well as retest and internal reliability, of VKC-2 automated scores.

**Results:**

A principal component analysis showed that the primary VKC-2 automated scores captured a single dimension and could be combined into a composite score reflecting task efficiency. Construct validity was supported by analyses of covariance results showing that participants with healthy cognition obtained significantly better VKC-2 scores than participants with cognitive impairment (all *Ps*<.001), even after controlling for demographics and general computer visuomotor dexterity. Convergent validity was supported by significant correlations between VKC-2 scores and performance on the Real Kitchen (r=−0.58 to 0.64, *Ps*<.001), conventional cognitive test scores (r=−0.50 to −0.22, *Ps*<.001), and self- and informant report questionnaires evaluating everyday function (r=0.25 to 0.43, *Ps*<.001). Intraclass correlation coefficients (ICCs) indicated moderate to excellent retest reliability (ICC=0.70-0.90) for VKC-2 scores after 4-6 weeks. Reliability improved in analyses including only participants who reported no change in cognitive status between time 1 and time 2 (n=123). Spearman-Brown correlations showed acceptable to good internal consistency between the VKC-2 tasks (breakfast and lunch) for all scores (0.77-0.84), supporting the use of total scores.

**Conclusions:**

The VKC-2 is an efficient, valid, and sensitive measure of everyday function for diverse older adults and holds promise to improve the status quo of functional assessment in aging, particularly when informants are unavailable or unreliable.

## Introduction

As the US population ages and interventions for Alzheimer disease and Alzheimer disease–related dementias become available [1], highly sensitive, objective, and efficient measures of functional abilities are needed for multiple purposes. Mild functional difficulties are among the strongest predictors of future cognitive decline and dementia [2-5]; thus, accurate measurement of functional ability will improve prognostic prediction and help identify the need for early intervention. Given that functional ability level is often the criterion that distinguishes mild cognitive impairment (MCI) from mild dementia, accurate assessment is critical for diagnostic decision-making [6,7]. According to the Food and Drug Administration, the approval of pharmacological treatments for dementia, even at the very early, presymptomatic stage, is contingent on demonstrating gains on meaningful measures of functioning [8]. Recently approved treatments have relied on composite measures such as the Clinical Dementia Rating-Sum of Boxes and the integrated Alzheimer’s Disease Rating Scale, but these measures require specialized training, are not readily deployable in typical clinical settings, and lack sensitivity to the earliest functional changes [9,10]. There exists a critical need for sensitive and efficient functional assessment tools that are clinically meaningful, psychometrically sound, and practically implementable across diverse health care settings [11,12]. We developed a nonimmersive virtual reality (VR) measure, the Virtual Kitchen Challenge—Version 2 (VKC-2), an objective, sensitive, efficient, and theoretically based tool for assessing everyday function in older adults to address the gaps in current functional assessments. Here we report results on VKC-2 validity and reliability in racially diverse, community-dwelling older adults with healthy cognition, MCI, or mild dementia.

Self/informant reports of everyday function, which are easy to administer and score, are the current standard method for functional assessment. When used with reliable, observant, and knowledgeable reporters, they generate useful information about how a person is functioning in everyday life [13-15]. In many circumstances, however, the accuracy of self and informant reports is uncertain. Their subjective nature makes them prone to over- or underreporting due to faulty cognitive abilities, psychological factors (eg, denial, depression, burden), or cultural beliefs [16]. Informant reports are often unavailable, as many older adults do not have a living spouse, nearby family members, or close friends. Even when available and willing, informants may have limited opportunities to observe daily functioning and may lack knowledge, particularly when functional difficulties are mild and may be masked by compensatory behaviors [17,18].

Another limitation of questionnaires is that older adults vary widely in the activities they perform and the contexts in which they perform them. For example, informant-reported difficulties with medication management may be profoundly different for an older adult managing a single prescription while residing in a small, highly organized home with her spouse versus an older adult taking dozens of medications while living alone in a large, cluttered house [19]. However, given identical clinical presentations and cognitive test scores suggesting mild cognitive decline, the latter patient would likely be diagnosed with clinical dementia if she were unable to independently manage her medications. Failure to account for context and task complexity confounds the informant report of everyday function and precludes clear comparisons of functional abilities across individuals.

Further, many questionnaires do not distinguish difficulties due to physical versus cognitive limitations [14], and if they do, it may be difficult for an informant to fully understand the nature of the functional difficulties, particularly because physical and cognitive limitations often co-occur [20-22]. Informant and self-reports also do not offer a detailed characterization of types of functional difficulties arising from different underlying cognitive problems (eg, slowing, disorganized actions vs omission of crucial task steps), which could offer insights into interventions for improving function and reducing the risk of future functional disability [23,24].

Performance-based measures of function address many of the limitations of questionnaires; they are objective, standardize task complexity and context, and allow for detailed analysis of behavior and systematic comparison across individuals. The Naturalistic Action Test (NAT), for example, is a performance-based test of everyday function with strong psychometric properties, normative data, and suggested cut scores for healthy cognition versus MCI versus mild dementia [23,25-35]. Scoring NAT performance for subtle inefficient errors, called micro-errors, has increased the sensitivity of NAT tasks for detecting mild difficulties with everyday tasks [35-38]. Results from performance-based tests, such as the NAT, with added sensitive scoring procedures, have demonstrated that (1) healthy older adults make more errors and require more time to complete everyday tasks than younger adults [36,37,39-43]; (2) people with MCI make more errors than healthy controls but fewer errors than individuals with dementia [32,35,44-46]; and (3) the ability to accurately and efficiently perform everyday tasks is moderately correlated with performance on cognitive tests [27,28,35,37,47] and informant report of everyday function [27,29,31,35,48]. Together, these findings and others [37,49-52] suggest that standardized performance-based assessment of function is valid and reliable.

Despite their objectivity, validity, and potential for rich characterizations of function, current performance-based tests require extraordinary effort, limiting their implementation and scalability. Scoring, particularly scoring for subtle errors and inefficiencies, is time-intensive and requires video recording, detailed scoring instructions, and trained coders. Although some performance-based tests may be scored quickly as pass/fail without video recording [40,53], such gross measures are less sensitive to mild difficulties (ie, MCI) [54], do not advance our understanding of the nature of functional problems [5,55,56], or still require considerable effort to administer. To streamline administration and scoring, a nonimmersive VR task called the Virtual Kitchen, modeled after the NAT, was developed. The original version of the Virtual Kitchen [57] required a mouse to move objects on a computer screen to complete a coffee-making task. Results showed that people with dementia accomplished fewer steps and made more errors than healthy controls on the Virtual Kitchen. Validity was also supported by significant correlations between Virtual Kitchen scores and performance of real tasks, cognitive tests, and informant reports of functioning [57].

Our team revised the original Virtual Kitchen [57] by implementing the following updates: (1) expanding the coffee task to include a more extensive breakfast; (2) adding a lunch task; (3) updating the graphics; and (4) transitioning from a mouse to a computer touchscreen to make interactions more natural [39]. We also added a brief training task to familiarize participants with the touchscreen interface. Automated scores were expanded to include measures computed based on interactions with the touchscreen to increase sensitivity (ie, number of screen interactions). Preliminary results from the revised task, which we called the Virtual Kitchen Challenge (VKC), demonstrated validity and good internal consistency [39]. The VKC automated scores have been validated against conventional cognitive tests in young adults [52] and against neuroimaging markers of cerebral vascular disease (white matter hyperintensities) in a small sample of community-dwelling older adults [48].

In this paper, we present the psychometric properties of the automated scores from the most recent revision of the Virtual Kitchen, the VKC-2. This version includes enhanced graphics and a more extensive basic familiarization task for practice and to obtain a score of participants’ digital visuomotor dexterity that may be used as a control measure. We evaluated construct and convergent validity as well as retest and internal reliability in a large, community-based sample of racially diverse older adults with healthy cognition, MCI, or mild dementia. Construct validity of the VKC-2 automated scores was evaluated in a known-group comparison (healthy cognition vs MCI vs mild dementia). Convergent validity was evaluated with correlations between automated VKC-2 measures and performance on the real versions of the VKC-2 tasks (Real Kitchen), demographically adjusted cognitive test scores, and conventional self/informant questionnaires of everyday function. Retest reliability was evaluated over a period of 4-6 weeks. Internal consistency was evaluated for the 2 VKC-2 tasks (breakfast and lunch).

## Methods

### Participants

Participants were recruited for an observational, longitudinal psychometric study designed to evaluate the psychometric properties of the VKC-2 (n=217; grant R01AG062503) or for a separate, smaller study on activity tracking (n=20; grant F31AG089944). Procedures for the baseline visit of both studies were the same, designed and conducted in accordance with the Helsinki Declaration, and approved by the Institutional Review Board at Temple University (institutional review board protocols 23116 and 29712). All participants and a knowledgeable informant signed informed consent forms, were compensated for their participation (US $50 for participants per session and US $25 for informants per session), and were assigned study numbers to protect their privacy when storing research records. At the end of the study, participants were also offered a research report with their cognitive test scores, if interested.

All participants were recruited from community outreach events, fliers, and referrals from neurology departments in Philadelphia, Pennsylvania, from September 2020 to June 2025. Inclusion and exclusion criteria were screened by phone, with only minor differences between the 2 studies. In both studies, participants were excluded for the following reasons: lifetime history of severe psychiatric disorder (eg, schizophrenia, bipolar disorder); nervous system infections or disorders (eg, epilepsy, brain tumor); current metabolic or systemic disorders (eg, B_12_ deficiency, renal failure, cancer); current moderate-severe depression; current moderate-severe anxiety symptoms; severe sensory deficits that would preclude visual detection or identification of common everyday objects used in the study or the ability to hear task directions (eg, blindness, total hearing loss); severe motor weakness that would preclude the use of everyday objects (eg, severe deformities or paralysis of both upper extremities); intellectual disability; and not being a fluent English speaker. The inclusion criteria for the larger study required participants to be at least 65 years old and have an available informant who could serve as a study partner. Informants were screened by phone for the following eligibility criteria: 18 years of age or older; fluent English speaker; available and willing to complete study questionnaires in person, by phone, or online; has daily contact with the participant; and reports knowledge of the participant’s daily functioning. Inclusion criteria for the second, smaller study required participants to be at least 55 years old and did not require a study informant/partner.

### Procedures

At the baseline visit (session 1), participants (N=237) completed informed consent, cognitive testing, the VKC-2, the real version of the VKC-2 tasks (ie, Real Kitchen), and questionnaires regarding demographic information, familiarity with the tasks used in the VKC-2, and their ability to perform activities in everyday life. The order of the Real Kitchen and VKC-2 was counterbalanced across participants to control for order effects. At session 1, informants completed questionnaires in person, online, or at home by mail. After reaching our target sample size (n=140) for retest reliability analyses (June 2024), participants were no longer requested to return for a second session 4-6 weeks after session 1 [58]. A total of 143 participants completed session 2, which included a brief interview (for both the participant and informant) regarding changes in cognition or health status (eg, medication changes, falls, illnesses, hospitalizations) since session 1, as well as repeat administration of the VKC-2 and Real Kitchen.

### Measures

#### Conventional Cognitive Tests

Cognitive tests were administered to characterize the sample, classify participants according to their cognitive status, and evaluate the convergent validity of the VKC-2. The cognitive testing protocol is described in Table 1. The protocol included 2 tests from 4 different cognitive domains to classify participants according to Jak/Bondi actuarial criteria [59,60] and clinical criteria originally proposed by Petersen [6] and McKhann [61]. Normative data from the Calibrated Neuropsychological Normative System [62] were used to enable raw score adjustments for sex, age, education, and IQ estimated by a test of reading/vocabulary. Such demographic adjustments are critical for confirming group membership in a diverse sample of older adults [63,64]. Further details on how tests were used for classifying cognitive abilities are provided in Multimedia Appendix 1.

**Table 1:** Cognitive tests administered at session 1.

Cognitive domain and test		Score(s)	Reference
**Premorbid intellectual functioning (IQ)**			
	Hopkins Reading Test	Estimated IQ	Schretlen et al [65]
**Global cognitive status**			
	Mini-Mental State Examination	Total correct	Folstein et al [66]
**Episodic memory**			
	Hopkins Verbal Learning Test—Revised^a,b^	Delayed free recall total correct	Brandt and Benedict [67]
	Brief Visual Memory Test—Revised^a^	Delayed free recall total correct	Benedict et al [68]
**Language**			
	Category (Animal) Fluency^a,b^	Total correct	Schretlen et al [62]
	Boston Naming Test—30 item^a^	Total correct	Goodglass and Kaplan [69]
**Executive function**			
	Trail Making Test—Part B^a,b^	Completion time	Reitan [70]
	Digit Span Backward^a^	Longest span	Wechsler [71]
**Processing speed**			
	Salthouse Letter Comparison^a,b^	Total correct	Salthouse [72]
	Salthouse Pattern Comparison^a,b^	Total correct	Salthouse [72]
**Attention**			
	Digit Span Forward^a^	Longest span	Wechsler [71]
	Trail Making Test A^a^	Completion time	Reitan [70]

^a^*t* scores from these tests were used for healthy versus mild cognitive impairment versus dementia classification.

^b^*t* scores from these tests were averaged to compute the modified Knight-Preclinical Alzheimer Cognitive Composite.

For the analysis of VKC-2 convergent validity, composite scores were computed by averaging demographically adjusted *t* scores from tests within each domain (eg, Episodic Memory, Language). A global cognitive composite was modeled after the Knight-Preclinical Alzheimer Cognitive Composite (modified [m]Knight-PACC) [73], which has been validated as a sensitive measure of early cognitive change due to neurodegenerative disease.

#### Virtual Kitchen Challenge-Version 2

The VKC-2 is a nonimmersive VR test of everyday function that requires participants to complete 2 everyday tasks (breakfast and lunch) by moving virtual objects using a touchscreen [39,57]. The VKC-2 tasks and objects were modeled after the NAT [25], an extensively studied and theoretically based performance-based test of everyday function that involves completion of familiar everyday tasks using real objects. The VKC-2 breakfast and lunch tasks were designed to be of comparable complexity and difficulty, with each task including 13 target objects and 4 distractor objects. For this study, the VKC-2 was administered on an MSI Creator Z16-A12UET laptop (12th Gen Intel Core i9 Processor) with a 16″ QHD+ (Quad High Definition Plus) (2560 × 1600), 120 Hz, IPS (In-Plane Switching)-level touchscreen display to maximize visibility and portability. Participants were instructed to use the index finger of their dominant hand to move and manipulate objects on the touchscreen.

**Figure 1:**
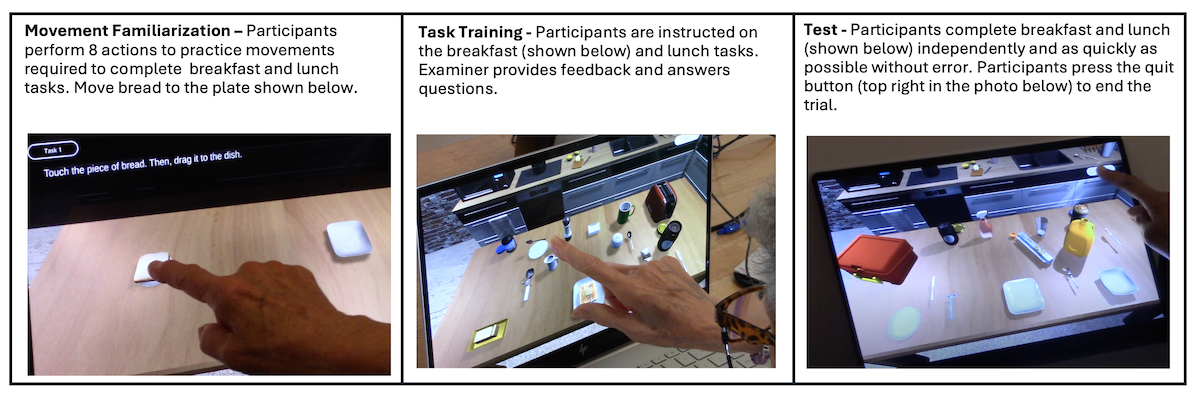
Photos of participants completing each phase of the VKC-2.

The VKC-2 included 3 phases: Movement Familiarization, Task Training, and Test. See Figure 1 and the text below for more details.

#### VKC-2 Movement Familiarization

Participants were directed to perform 8 basic touchscreen actions (eg, tap, drag) to complete the following task steps: (1) move bread to dish, (2) stir mug with spoon, (3) pour juice, (4) place thermos in lunch box, (5) spread jelly on bread, (6) wrap cookies in foil, (7) place bread in toaster, and (8) add sugar to mug. Participants first performed all basic touchscreen actions with guidance from the examiner and had the opportunity to ask questions and repeat each action as needed. Next, participants were asked to complete all 8 trials independently as quickly and efficiently as possible. Completion time of the second, independent trial was computed as a measure of basic digital visuomotor dexterity (Digital Dexterity Score).

#### VKC-2 Task Training

The examiner reviewed the written instructions presented on the computer screen for each task. Participants were asked to point to each of the target objects needed for the task. For example, training for the breakfast task included the direction to “point to all of the objects you will need for the toast” while the examiner named each object out loud (eg, “bread,” “toaster”). Participants were also asked to point to each of the distractor objects and were told that they would not need to touch or use those objects. Participants then proceeded to practice trials, making breakfast and lunch with prompting, cues, and error correction from the examiner. The examiner also answered questions to ensure that participants fully understood each task.

#### VKC-2 Test

Breakfast and lunch tasks were completed independently without feedback. Instructions regarding the task objectives, which were reviewed during the practice trials, were repeated (eg, “pack a lunch for someone who wants a sandwich, snack, and a drink”). Participants were also instructed to complete test trials as quickly as possible, without making errors, and using clear and precise movements. They were told to touch the quit button at the top right of the screen to end the trial (see Figure 1). Participants were asked to verbally repeat the directions before each task to ensure comprehension; instructions were repeated as often as needed before the participant initiated the task.

#### VKC-2 Test Automated Scores

Performance on the VKC-2 Test tasks (breakfast and lunch) was scored using data from the touchscreen, as described and validated in our pilot work with the original version of the VKC [39,48,52]:

- Completion time (time) was recorded in seconds from the moment the virtual kitchen screen appeared (after instructions) until the participant pressed the quit button. Results from prior studies of the original VKC indicate that completion time differed significantly between older and younger participants and correlated with completion time on the Real Kitchen, cognitive tests of executive function and episodic memory [39], and neuroimaging markers of cerebrovascular disease [48].
- The number of screen interactions (touches) included the number of discrete instances the participant made contact with the computer touchscreen. Touches were collected as a measure of performance efficiency, with fewer screen interactions reflecting more precise and deliberate actions. Results from the original VKC showed that older adults made significantly more touches than younger adults, with additional touches by older adults including both inefficient correct actions and errors. A higher number of touches was significantly associated with more total errors scored by trained coders who watched video recordings of VKC performance. Additionally, screen touches were significantly associated with performance on the Real Kitchen and cognitive tests of executive function and episodic memory [39].
- The percentage of time off-screen (%off-screen) was the percentage of time spent working on the VKC-2 when the participant was not touching the screen. It was computed by subtracting the time spent touching the screen from the completion time, dividing by the completion time, and multiplying by 100. The percentage of time off-screen also reflects performance efficiency. Pilot data from the original VKC [39] indicated that older adults spent a significantly higher percentage of their total time off-screen than younger adults. Correlations between %off-screen and human codes of VKC performance suggested that higher %off-screen times were due to multiple factors, including slower planning, difficulties locating target objects, difficulty resolving competition for object selection, and misreaching toward the computer screen (ie, micro-errors). Higher %off-screen times were significantly associated with more errors on the Real Kitchen, poorer scores on tests of executive function [52] and episodic memory [39], and neuroimaging markers of cerebrovascular disease [48].
- The number of distractor object interactions (distractor interactions) included instances when a distractor object was touched or moved. Our pilot work in a sample of healthy older and younger adults indicated that distractor interactions occurred too infrequently for analysis [39], but they have not been studied in participants with cognitive impairment.

#### Real Kitchen

**Figure 2:**
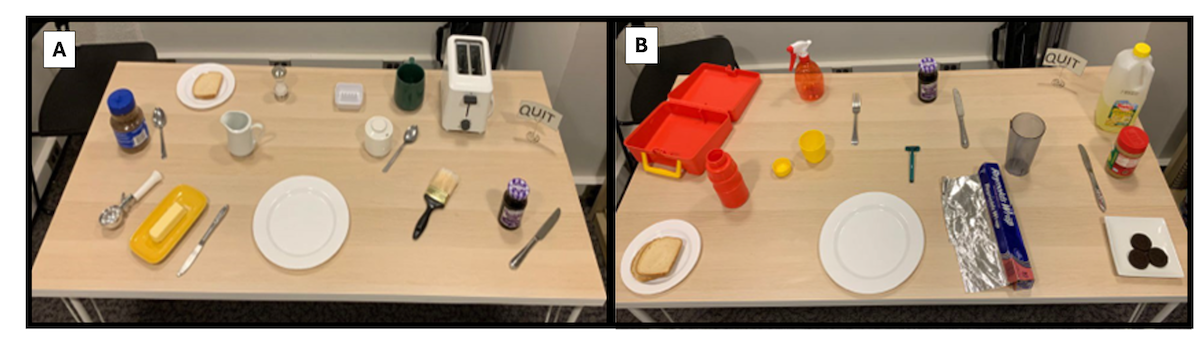
Real Kitchen breakfast (A) and lunch (B) tasks.

The Real Kitchen required participants to complete the breakfast and lunch tasks using real objects placed on a table (Figure 2). Instructions for the Real Kitchen were identical to those for the VKC-2, including the instruction to “press the quit button when finished.” In the Real Kitchen, the Quit Button was a piece of paper on the right side of the table labeled “QUIT.” Real task objects were similar in appearance (color and shape) to the simulated objects in the VKC-2. Participants repeated the directions before each test trial to ensure comprehension; instructions were repeated as often as needed. Participants were video recorded, and recordings were labeled using a code so that human coders were unaware of participant classification and study session.

Real Kitchen performance was scored according to detailed instructions using validated scores and procedures. Real Kitchen scores from a subset of the current sample have been published and show strong interrater reliability, significant differences between participants with healthy cognition versus cognitive impairment, and correlations with cognitive tests and self/informant reports of everyday function [35]. For our current study, the following Real Kitchen scores were used to validate (convergent validity) the VKC-2 automated measures:

- Real Kitchen completion time was recorded in seconds and reliably coded by starting the timer when the first step was initiated and ending when the participant touched the quit button. Prior work shows that participants with greater cognitive impairment demonstrate longer completion times than participants with healthy cognition [35].
- Accomplishment was coded for each completed step and scored from 0 to 13 for the breakfast task and 0 to 20 for the lunch task. A total accomplishment score was computed (0-33), with higher scores reflecting a greater number of task steps accomplished.
- Total errors were coded according to a taxonomy studied in a range of clinical populations [25,74], showing validity and strong interrater reliability in people with stroke [75,76], dementia [27,28,30,47], MCI [26,32,33], and healthy controls [37,38,49], as well as a subset of participants from this sample [35]. The error taxonomy (see Multimedia Appendix 1) includes overt errors (eg, performing task steps in the wrong sequence) and micro-errors (eg, reaching toward a distractor object). In studies of participants with dementia, total overt errors correlate with cognitive tests and informant reports of function. The micro-error category was added to improve detection of subtle, inefficient behaviors in healthy and MCI participants [35,37,38,49]. As overt errors occur with relatively low frequency, they were combined with micro-errors to compute a total error score [35].
- Motor errors were tracked separately from total errors. Motor errors involved instances in which a corrective action was performed with motor or spatial imprecision (eg, spilling coffee grounds, dropping a knife).

#### Participant Questionnaires

Participants completed a demographic form assessing age, sex, race, ethnicity, income, and education level, as well as the following questionnaires.

The Past Experience Scale [45,77] assessed familiarity with the breakfast and lunch subtasks that comprise the VKC/Real Kitchen. The scale included 4 items (toast, coffee, sandwich, and thermos), each rated from 0 (not at all familiar) to 4 (very familiar). The total familiarity score ranged from 0 to 16, with higher scores reflecting greater familiarity. Participants also rated the frequency with which they had completed each subtask in their day-to-day life over the past 5-10 years, using a scale from 0 (never) to 4 (just about every day), with total scores ranging from 0 (never performed any of the tasks) to 16 (performed each task just about every day).

Functional Activity Questionnaire (FAQ) [14] instructions were modified to reflect only difficulties due to cognitive problems (not physical problems, fatigue, etc) for 10 activities (eg, preparing a balanced meal). Each activity is rated on a scale from 0 (performs normally) to 3 (dependent). Total FAQ scores range from 0 to 30, with higher scores reflecting greater dependence on others in everyday tasks due to cognitive difficulties.

The 12-item Everyday Cognition Scale (ECog-12) [13,78] measures decline over the past 10 years in 12 everyday cognitive abilities (eg, remembering where you have placed objects) on a scale from 1 (better or no change) to 4 (much worse all the time). Total scores reflect an average across all completed items and range from 1 to 4, with higher scores indicating greater decline in everyday cognition.

The Instrumental Activities of Daily Living—Compensation (IADL-C) [15] scale measures the need for assistance and compensatory strategies when performing 27 daily activities (eg, preparing one’s own meals). Each activity is rated on a scale from 1 (independent, no aid) to 8 (not able to complete the activity anymore). The total score is the sum of all item responses, with a possible range from 27 (completely independent, no aid needed for any tasks) to 216 (no longer able to perform any task).

#### Informant Questionnaires

Informants completed questionnaires regarding their demographic information (eg, age, education), their relationship with the participant (eg, cohabitation, years known, hours in contact with the participant), and the participants’ everyday function, including the ECog-12 [13,78], FAQ [14], and IADL-C [15]. Instructions and scoring for each questionnaire were the same as those for the participant versions described above.

### Analysis Plan

#### Preliminary Analysis

Analyses were conducted using SPSS version 29.0 (IBM Corp) [79]. VKC-2 automated scores were examined for outliers and Winsorized at the first and ninety-ninth percentiles. The VKC-2 distractor interaction score was dichotomized because a few participants interacted with distractor objects (0=no interactions; 1=at least one interaction with a distractor object during completion of the VKC-2). Relations among VKC-2 scores were evaluated using bivariate correlations. Additionally, a principal component analysis (PCA) was conducted, including the 3 primary VKC-2 variables (time, touches, and %off-screen), to determine whether the dimensional VKC-2 automated scores could be combined into a single composite score. The Digital Dexterity score was not included in the PCA because it is derived from a separate condition intended to be used as a control for basic visuomotor skills. The distractor interaction score was not included because dichotomous variables are not appropriate for PCA. The suitability of the data for PCA was evaluated using the Kaiser-Meyer-Olkin measure of sampling adequacy and the Bartlett test of sphericity.

#### Construct Validity

VKC-2 automated scores were compared across groups known to differ in functional ability level: healthy cognition, MCI, and mild dementia. As the size of the dementia subgroup was relatively small (n=16), statistical analyses focused on differences between participants with healthy cognition and those with MCI. Participants with dementia were included for descriptive comparisons. One-way analyses of covariance (ANCOVA) were used to test group differences for each VKC-2 automated score (digital dexterity, time, touches, %off-screen, and VKC-2 composite) after controlling for demographics. Group differences were also evaluated in ANCOVA models that controlled for the digital dexterity score to determine whether significant group differences were explained by differences in basic visuomotor or computer abilities. Group differences on the dichotomized VKC-2 distractor interaction score were evaluated using chi-square tests. Significant between-group differences with at least small effect sizes (ie, partial η^2^>.01; phi [φ] coefficient>.30) were interpreted as supporting the construct validity of the VKC-2 automated scores.

Receiver operating characteristic analyses comparing participant groups (healthy cognition vs impaired cognition [MCI + dementia]; healthy cognition vs MCI) were performed to identify cutoff values for each of the VKC-2 automated scores. Youden indices were used to identify cutoff scores that optimized sensitivity and specificity [80].

#### Convergent Validity

Correlations between the VKC-2 automated measures and the ability to perform tasks with real objects (Real Kitchen), demographically adjusted cognitive test scores of overall cognition and specific cognitive abilities, and self/informant reports of everyday functioning were performed to evaluate convergent validity. Pearson correlation coefficients were computed using the full sample. Spearman rank-order correlations were also performed and are included in Tables S3-S5 in Multimedia Appendix 1. Significant and moderate-level relationships were interpreted as supporting the convergent validity of the VKC-2 automated scores.

#### Reliability

Retest reliability was assessed using intraclass correlation coefficients (ICCs), calculated with a 2-way mixed-effects model based on absolute agreement and average measures [81]. ICC values range from 0 to 1, with values above 0.75 generally indicating good reliability and values above 0.90 considered excellent [82]. 95% CIs were computed for each ICC, and significance was determined using *F* tests. Retest reliability for the distractor interaction score (dichotomous variable) was examined using Cohen κ [83]. Retest reliability was evaluated for the full sample who completed session 2 (n=143) and for a subsample that reported no change in cognitive abilities since session 1 (123/143). Internal consistency between the 2 VKC-2 tasks (breakfast and lunch) was tested using the Spearman-Brown formula (*r*), with coefficients>0.70 interpreted as evidence of strong internal consistency [84].

## Results

### Participant Characteristics

A total of 237 participants were recruited from June 2021 to June 2025 for studies on everyday function. One participant with mild dementia refused to complete the study tasks; thus, the final analytic sample included 236 participants, of whom 172 were classified as having healthy cognition, 48 as having MCI, and 16 as having mild dementia. On average, participants were 72 years old and had completed 15 years of education; of the 236 participants, 156 (66.1%) were women, and nearly equal numbers identified as Black (n=106, 44.9%) and White (n=113, 47.9%). Demographic characteristics of the groups are reported in Table 2. The groups differed in age and education, but post hoc comparisons did not reach statistical significance (*P*>.051 for all). There were no group differences in estimated IQ or in the distributions of sex, Black/African American versus White race, or ethnicity.

**Table 2:** Demographic and descriptive characteristics by group.

Variable			Healthy (n=172)		Mild cognitive impairment (n=48)		Mild dementia (n=16)		*F* test (*df*) or chi-square (*df*)		*P* value
Age, mean (SD); range			71.95 (6.56); 58-94		74.54 (7.27); 61-98		74.50 (8.70); 55-91		3.30 (2, 235)		.04
Education (years), mean (SD); range			16.06 (2.51); 10-20		15.40 (3.25); 10-20		14.06 (3.04); 10-20		4.64 (2, 235)		.01
Estimated IQ, mean (SD); range			112.44 (11.73); 87-139		112.06 (13.30); 88-138		108.25 (13.19); 88-139		0.87 (2, 235)		.42
Sex: women, n (%)			114 (66.3)		33 (68.8)		9 (56.3)		0.85 (2)		.67
**Race**									3.53 (2)		.17
	Black	72 (41.9)		26 (54.2)		8 (50.0)					
	White	89 (51.7)		17 (35.4)		7 (43.8)					
	Asian	5 (2.9)		3 (6.3)		1 (6.3)					
	Pacific Islander/Hawaiian	2 (1.2)		0 (0)		0 (0)					
	American Indian	0 (0)		1 (2.1)		0 (0)					
	Multiracial	3 (1.7)		1 (2.1)		0 (0)					
	Not reported	1 (0.6)		0 (0)		0 (0)					
Latino/Hispanic Ethnicity			2 (1.2)		0 (0)		0 (0)		0.75 (2)		.69
**Past Experience Scale**											
	Familiarity rating, mean (SD); range	14.20 (2.44); 6-16		13.45 (3.27); 3-16		10.80 (4.44); 4-16		10.69 (2, 235)		<.001	
	Frequency rating, mean (SD); range	7.87 (2.82); 0-16		7.89 (3.27); 2-13		8.27 (1.62); 5-11		1.11 (2, 235)		.87	

Results from the Past Experience Scale showed that task familiarity ratings were generally high, indicating that, on average, the breakfast and lunch tasks were “pretty” to “very” familiar. The groups differed on the familiarity rating, with post hoc tests indicating that the dementia group reported significantly lower task familiarity than the healthy cognition group (*P*<.001) and the MCI group (*P*=.005); however, the healthy cognition group and the MCI group did not differ (*P* =.32). According to the frequency ratings, participants reported that, on average, they had performed the VKC-2 tasks about once per month over the past 5-10 years. Frequency ratings did not differ across groups.

Demographic characteristics of participants who returned for session 2 and were included in the retest reliability analysis are reported in Table S1 in Multimedia Appendix 1. Compared with participants who did not return, the returning participants had completed significantly more years of education, obtained higher estimated IQ scores, and included a greater proportion of White participants.

### Informant Characteristics

A total of 219 informants participated in the study. On average, informants were 63.97 years old (SD 13.99 years; range 20-90 years) and had completed 15.73 years of education (SD 2.43 years; range 10-21 years). Informants included spouses (95/219, 43.4%), children (60/219, 27.4%), friends (41/219, 18.7%), and other family members (23/219, 10.5%).

### Correlations Among VKC-2 Scores and Principal Component Analysis

Average VKC-2 scores and their bivariate correlations indicate significant, moderate associations among all scores (Table 3). The relationship between VKC-2 time and touches was particularly strong, reflecting nearly overlapping scores, with more touches associated with longer completion times.

**Table 3:** VKC-2^a^ scores and correlation coefficients in the full sample (N=236).

VKC-2 score	Digital dexterity	Time	Touches	%Off-screen
Time	0.67^b^	N/A^c^	N/A	N/A
Touches	0.51^b^	0.83^b^	N/A	N/A
%Off-screen	0.49^b^	0.42^b^	0.34^b^	N/A
Distractor interactions	0.34^b^	0.39^b^	0.42^b^	0.26^b^
Mean	86.76	197.47	67.82	0.48
SD	27.47	111.57	50.59	0.09

^a^VKC-2: Virtual Kitchen Challenge—Version 2.

^b^*P*<.001 (2-tailed). A total of 21 (8.9%) participants interacted with distractor objects; the mean and SD for the distractor interactions score are not reported because it was dichotomized.

^c^N/A: not applicable.

According to the Kaiser-Meyer-Olkin measure of sampling adequacy (0.579) and the significant Bartlett test of sphericity (*χ*^2^_3_=311.45, *P*<.001), there was a modest but acceptable level of shared variance among variables and a suitable correlation matrix for factor analysis. PCA results showed that only 1 component was extracted (eigenvalue=2.09), accounting for 69.74% of the total variance. All variables loaded positively on this component (time=0.930, touches=0.905, and %off-screen=0.639), suggesting a single underlying factor representing a common dimension. Thus, a VKC-2 composite score was computed by averaging sample-based *z* scores for time, touches, and %off-screen, with higher scores reflecting worse (ie, more inefficient) performance.

### Construct Validity

**Figure 3:**
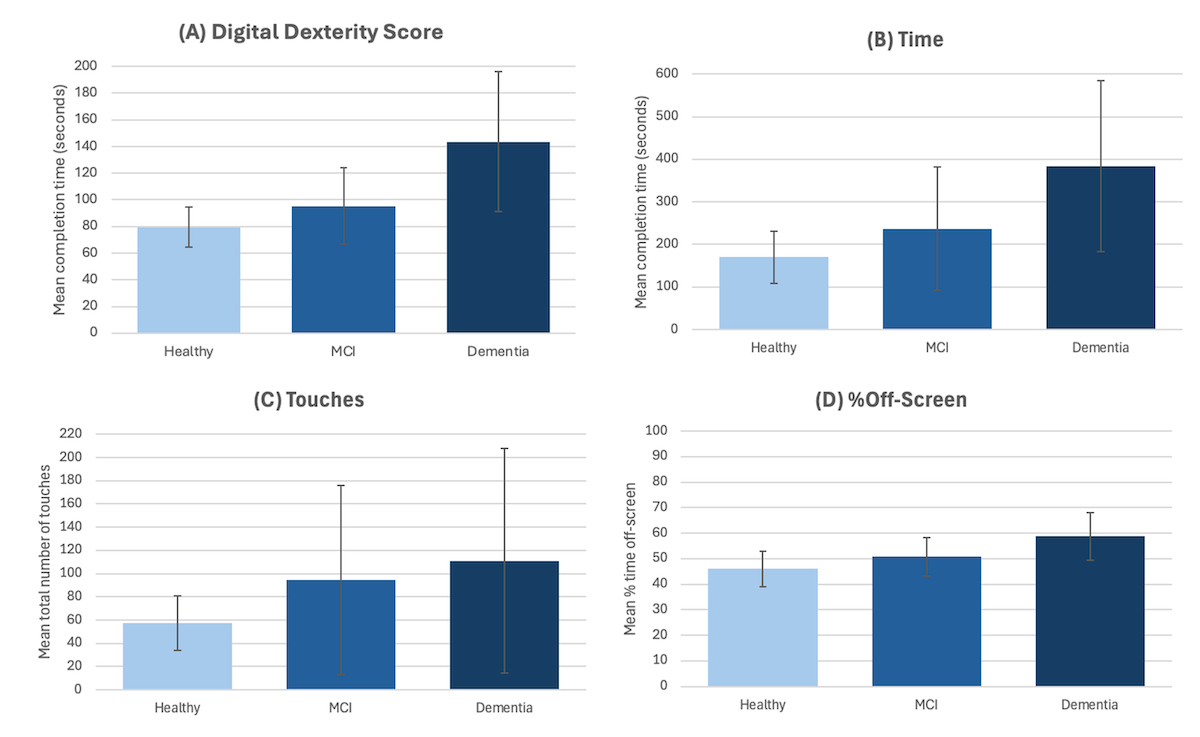
Unadjusted VKC-2 mean scores by group.

The construct validity of the VKC-2 automated scores was evaluated by assessing differences among groups known to differ in functional abilities: healthy, MCI, and mild dementia. As shown in Figure 3, average scores on each VKC-2 measure were consistently worse for the dementia group. The same pattern was observed in the VKC-2 composite score (healthy: mean –0.22, SD 0.49; MCI: mean 0.43, SD 1.07; and dementia: mean 1.27, SD 1.36). Statistical analyses focused on differences between the healthy and MCI groups due to the relatively small number of participants with dementia. ANCOVA results comparing healthy versus participants with MCI are reported in Tables 4 and 5 and showed significant group differences (*P*<.001) in all measures after controlling for age. After controlling for the digital dexterity score and age (see Tables 4 and 5), the difference in the time score was no longer significant (*P*=.06), suggesting that the difference in completion time could be explained by low-level visuomotor skill differences between the MCI and healthy groups. By contrast, after controlling for digital dexterity and age, the differences in touches (*P*=.004), %off-screen (*P*=.01), and the VKC-2 (*P*<.001) composite score remained statistically significant, indicating that these between-group differences could not be explained by basic visuomotor skills. Thus, aside from time, the VKC-2 scores—particularly the composite score, which showed the strongest effect size after controlling for digital dexterity and age—likely reflect more than simple visuomotor abilities and capture the cognitive processes required to perform everyday tasks (ie, goal maintenance and control over task goals for the efficient execution of multistep everyday tasks).

**Table 4:** Analysis of covariance results comparing participants with healthy cognition (n=172) versus those with mild cognitive impairment (n=48) on all VKC-2^a^ automated scores: controlling for age.^b^

VKC-2 score	*F* test (*df*)	*P* value	η² (partial eta^2^)	Effect size
Digital dexterity	20.68 (2, 219)	<.001	0.087	Medium to large
Time	17.54 (2, 219)	<.001	0.075	Medium
Touches	23.35 (2, 219)	<.001	0.097	Large
%Off-screen	12.71 (2, 219)	<.001	0.055	Medium
VKC-2 composite	29.70 (2, 219)	<.001	0.120	Medium to large

^a^VKC-2: Virtual Kitchen Challenge—Version 2.

^b^Effect sizes (η^2^) are interpreted as follows: small=0.01, medium=0.06, and large=0.14.

**Table 5:** Analysis of covariance results comparing participants with healthy cognition (n=172) versus those with mild cognitive impairment (n=48) on all VKC-2^a^ automated scores: controlling for age and digital dexterity.^b^

VKC-2 score	*F* test (*df*)	*P* value	η² (partial eta²)	Effect size
Time	3.47 (3, 219)	.06	0.016	Small
Touches	8.60 (3, 219)	.004	0.038	Small to medium
%Off-screen	6.42 (3, 219)	.01	0.029	Small to medium
VKC-2 composite	11.68 (3, 219)	<.001	0.051	Medium

^a^VKC-2: Virtual Kitchen Challenge—Version 2.

^b^Effect sizes (η^2^) are interpreted as follows: small=0.01, medium=0.06, and large=0.14.

The distributions of the distractor interaction score across the 3 groups (not reported in Figure 3) indicated a higher percentage of participants interacting with distractors in the groups with cognitive impairment (dementia: 5/16, 31%; MCI: 8/48, 17%; and healthy: 5/114, 4.3%). The difference in distractor interactions between the MCI and healthy groups was statistically significant (*χ*^2^_1_=8.03, *P*=.005; φ=0.191, small-to-medium effect size).

### Classification Analyses

Classification analyses for distinguishing participants with healthy cognition from those with cognitive impairment (MCI + mild dementia combined) are reported in Table 6. All predictors showed statistically significant areas under the curve (AUCs; *P*<.001 for all), indicating that they were better than chance at predicting impaired group status. Time and the VKC-2 composite score were the strongest predictors, as indicated by their high AUCs and sensitivity. Time demonstrated particularly high sensitivity, making it useful for maximizing the identification of participants with impairment for early detection. By contrast, the %off-screen score showed the highest specificity, suggesting it may be more useful for ruling out individuals with healthy cognition during diagnostic confirmation. As expected, scores that increase sensitivity reduce specificity, reflecting the inherent trade-off between identifying true positives and minimizing false positives. Analyses distinguishing participants with healthy cognition versus those with MCI demonstrated similar AUCs (0.68-0.74), cutoff scores, and patterns of sensitivity and specificity, and are reported in Table S2 in Multimedia Appendix 1.

**Table 6:** Area under the curve values, optimal cutoffs, and specificity/sensitivity for predicting cognitive impairment from VKC-2^a^ scores (N=236).

VKC-2 score	AUC^b^	95% CI	SE	*P* value	Optimal cutoff	Sensitivity	Specificity
Digital dexterity	0.73	0.65-0.81	0.41	<.001	87.12	0.67	0.77
Time	0.75	0.68-0.82	0.04	<.001	163.47	0.82	0.58
Touches	0.70	0.62-0.78	0.04	<.001	65.5	0.59	0.78
%Off-screen	0.71	0.64-0.79	0.04	<.001	0.53	0.48	0.87
VKC-2 composite	0.76	0.70-0.84	0.04	<.001	–0.041	0.69	0.72

^a^VKC-2: Virtual Kitchen Challenge—Version 2.

^b^AUC: area under the curve.

### Convergent Validity Against Real Kitchen Scores

Bivariate correlations between VKC-2 scores and Real Kitchen scores are reported in Table 7. Correlations with Real Kitchen completion time, accomplishment, and total errors were consistently significant (*P*<.001 for all) and moderate to strong, supporting the convergent validity of the VKC-2 scores against the real versions of the VKC-2 tasks. Relations between VKC-2 measures and motor errors on the Real Kitchen were relatively weaker and not consistently significant (*P* values ranged from <.001 to .08), suggesting that VKC-2 scores correspond more strongly with the cognitive aspects of Real Kitchen performance rather than visuomotor errors made with the real tasks (Table 7). Spearman rank-order correlations showed the same pattern of results and are reported in Table S3 in Multimedia Appendix 1.

**Table 7:** Correlation coefficients (and P values) between VKC-2^a^ scores and Real Kitchen scores (n=201).

VKC-2 score	Completion time	Accomplishment score	Total errors	Motor errors
Digital dexterity	0.59^b^	–0.58^b^	0.50^b^	0.13 (*P*=.08)
Time	0.58^b^	–0.53^b^	0.64^b^	0.22 (*P*=.004)
Touches	0.38^b^	–0.26^b^	0.53^b^	0.30^b^
%Off-screen	0.44^b^	–0.44^b^	0.40^b^	0.15 (*P*=.057)
VKC-2 composite	0.56^b^	–0.48^b^	0.62^b^	0.27^b^
Mean	244.35	32.09	7.42	2.37
SD	93.73	2.35	5.88	2.49

^a^VKC-2: Virtual Kitchen Challenge—Version 2.

^b^*P*<.001 (2-tailed).

### Convergent Validity Against Conventional Cognitive Tests

Bivariate correlations between VKC-2 scores and demographically adjusted cognitive test scores are reported in Table 8. The coefficients were statistically significant (*P* values ranged from <.001 to .03), indicating that participants with higher cognitive test scores completed the VKC-2 tasks more quickly and efficiently, supporting the convergent validity of the VKC-2 scores. Spearman rank-order correlations showed the same pattern of results and are reported in Table S4 in Multimedia Appendix 1.

**Table 8:** Correlation coefficients (and P values) between VKC-2^a^ scores and cognitive test scores (N=236).

VKC-2 score	Global Cognition mKnight-PACC^b^	Executive function composite	Episodic memory composite	Processing speed composite	Language composite
Digital dexterity	–0.50^c^	–0.29^c^	–0.42^c^	–0.41^c^	–0.36^c^
Time	–0.42^c^	–0.27^c^	–0.39^c^	–0.30^c^	–0.34^c^
Touches	–0.23^c^	–0.14 (*P*=.03)	–0.29^c^	–0.11 (*P*=.10)	–0.22^c^
%Off-screen	–0.38^c^	–0.27^c^	–0.40^c^	–0.26^c^	–0.27^c^
VKC-2 composite	–0.42^c^	–0.27^c^	–0.42^c^	–0.28^c^	–0.34^c^
Mean	50.50	49.15	45.04	53.08	47.97
SD	7.66	8.77	10.04	9.75	9.55

^a^VKC-2: Virtual Kitchen Challenge—Version 2.

^b^mKnight-PACC: modified Knight-Preclinical Alzheimer Cognitive Composite.

^c^*P*<.001 (2-tailed).

### Convergent Validity Against Self/Informant Questionnaires of Everyday Function

Table 9 shows the relationships between VKC-2 scores and questionnaires assessing everyday function completed by participants and informants. Results from participant questionnaires indicated that the associations between VKC-2 scores and the IADL-C and FAQ, which assess current functional abilities, were statistically significant (*P* values ranged from <.001 to .02) and in the expected direction. That is, participants who reported greater current functional difficulties (IADL-C and FAQ) also performed the VKC-2 tasks less quickly and efficiently. The relationship between VKC-2 scores and participants’ reports of functional decline (ECog-12) was not significant (*P* values ranged from .21 to .55). By contrast, informant reports of both current functional difficulties (IADL-C and FAQ) and functional decline (ECog-12) were significantly associated with lower VKC-2 scores. Overall, correlations between the VKC-2 and participant/informant questionnaires support the validity of the VKC-2 and are comparable to or stronger than the relationships reported between conventional performance-based tests and questionnaires in the literature [43]. Spearman rank-order correlations showed a similar pattern of results and are reported in Table S5 in Multimedia Appendix 1.

**Table 9:** Correlation coefficients (and P values) between VKC-2^a^ scores and questionnaires.

VKC-2 score	Participant questionnaires (n=236)				Informant questionnaires (n=194)		
	IADL-C^b^	FAQ^c^	ECog-12^d^	IADL-C		FAQ	ECog-12
Digital dexterity	0.26^e^	0.32^e^	0.08 (*P*=.26)	0.43^e^		0.32^e^	0.34^e^
Time	0.28^e^	0.28^e^	0.06 (*P*=.38)	0.41^e^		0.32^e^	0.26^e^
Touches	0.15 (*P*=.03)	0.20 (*P*=.003)	0.04 (*P*=.55)	0.20 (*P*=.005)		0.10 (*P*=.17)	0.07 (*P*=.35)
%Off-screen	0.26^e^	0.25^e^	0.09 (*P*=.21)	0.35^e^		0.29^e^	0.31^e^
VKC-2 composite	0.27^e^	0.30^e^	0.07 (*P*=.30)	0.37^e^		0.28^e^	0.24^e^
Mean	44.76	2.12	1.56	46.75		2.92	1.40
SD	21.41	3.85	.97	30.10		5.98	.54

^a^VKC-2: Virtual Kitchen Challenge—Version 2.

^b^IADL-C: Instrumental Activities of Daily Living—Compensation.

^c^FAQ: Functional Activity Questionnaire.

^d^ECog-12: 12-item Everyday Cognition Scale.

^e^*P*<.001 (2-tailed).

### Retest Reliability

ICCs are reported in Table 10 and indicate moderate to excellent reliability for the VKC-2 automated scores. Cohen κ, used to assess agreement between distractor interaction scores at time 1 and time 2, showed only fair agreement (κ=0.27, *P*<.001), indicating limited but statistically significant consistency over time. When ICCs were rerun, including only participants who reported no change in their cognitive status from session 1 (123/143, 86%), results yielded comparable or slightly improved coefficients relative to the full sample (see Table S6 in Multimedia Appendix 1).

**Table 10:** Intraclass correlation coefficients for VKC-2^a^ scores over time (n=143).

VKC-2 score	Intraclass correlation coefficient^b^ (average measures)	95% CI	*F* test (*df*)	*P* value
Digital dexterity	0.844	0.766-0.893	6.965 (142, 142)	<.001
Time	0.812	0.736-0.865	5.469 (142, 142)	<.001
Touches	0.849	0.783-0.893	6.943 (140, 140)	<.001
%Off-screen	0.703	0.523-0.806	3.837 (140, 140)	<.001
VKC-2 composite	0.899	0.860-0.923	9.965 (140, 140)	<.001

^a^VKC-2: Virtual Kitchen Challenge—Version 2.

^b^Type A using an absolute agreement definition.

### Internal Reliability

Internal consistency between the VKC-2 breakfast and lunch tasks at time 1 was evaluated using Spearman-Brown coefficients in the full sample (N=236). Results indicated acceptable to good internal consistency for all scores (time: 0.81; touches: 0.81; %off-screen: 0.77; and VKC-2 composite score: 0.84).

## Discussion

Results of this study support the validity and reliability of the VKC-2 automated scores as measures of everyday function in older adults. As predicted, VKC-2 scores differed significantly between groups known to vary in functional ability (healthy vs MCI vs mild dementia), supporting the construct validity of the VKC-2. Convergent validity was further supported by significant correlations between VKC-2 scores and performance on the real versions of the VKC-2 tasks (Real Kitchen), conventional cognitive test scores, and self/informant questionnaires assessing everyday functioning. Retest reliability analyses showed fair to excellent reliability for the VKC-2 automated scores over 4-6 weeks. Internal consistency between the 2 VKC-2 tasks (breakfast and lunch) was also good. Additionally, participants reported that the tasks included in the VKC-2 were highly familiar (Past Experience Questionnaire). These findings suggest that the VKC-2 automated scores hold strong potential for addressing critical gaps in functional assessment across multiple contexts, including screening older adults at risk for decline in meaningful everyday activities in primary care and serving as a functional end point in clinical trials of Alzheimer disease/Alzheimer disease–related disorder treatments.

To our knowledge, this is the first study to demonstrate significant differences between older adults with healthy cognition and those with MCI on the VKC-2 automated scores. Group differences in all scores except time persisted even after controlling for the digital dexterity score, a novel feature of the updated VKC-2. Thus, differences between MCI and healthy participants on the VKC-2 cannot be attributed solely to differences in digital visuomotor skills or touchscreen accuracy, but rather reflect the additional cognitive demands required to perform everyday tasks accurately and efficiently (eg, accurate object selection, sequencing of task steps, performance monitoring [74,85,86]). This conclusion is further supported by significant correlations with Real Kitchen scores (see also [57,87]) and by the fact that differences between participants with MCI and healthy participants on the VKC-2 mirror those observed on performance-based tasks with real objects in previous studies [26,32,33,35,45]. Significant associations with cognitive tests of episodic memory and language, which do not primarily measure motor skills or processing speed, provide additional evidence that the automated VKC-2 scores reflect cognitive abilities. Collectively, these results strongly support the construct validity of the VKC-2, offering a novel approach to identify everyday task difficulties without the need for video recording or trained coders—a major advantage over traditional performance-based tests—providing a highly efficient, scalable, and sensitive measure of everyday functioning.

It is important to acknowledge that some VKC-2 scores reflect visuomotor skills more than others. For example, the completion time (time) score did not remain significantly different between participants with MCI and those with healthy cognition after controlling for the digital dexterity score. This should not be viewed as a limitation, as mild upper motor dexterity difficulties contribute to functional impairments in people with MCI [20], and mild upper and lower limb difficulties are significantly associated with cognitive challenges in older adults without MCI [21,22,88]. Indeed, the VKC-2 digital dexterity score, as well as VKC-2 measures of efficiency, were associated with a measure of global cognitive abilities (mKnight-PACC) that is sensitive to preclinical Alzheimer disease. Thus, mild motor difficulties may serve as important early indicators of Alzheimer disease/Alzheimer disease–related disorder risk that could be missed by conventional cognitive tests. Additional studies, including longitudinal follow-up, are needed to identify the optimal combination of VKC-2 scores to maximize early detection of functional difficulties and risk.

Correlation analyses with conventional self- and informant-report questionnaires of everyday function provided additional support for the validity of the VKC-2 automated measures as indicators of processes that influence real-world functioning. The strength and pattern of correlations between VKC-2 scores and conventional questionnaires were similar to those reported for validated performance-based tests and questionnaires of everyday function in the existing literature [89,90]. Correlations were stronger and more consistent with informant reports than with self-reports, particularly for the questionnaire assessing cognitive/functional decline (ECog-12 [13]); this pattern has been reported in previous studies [56] and aligns with our conceptualization of the constructs measured by performance-based tests versus questionnaires. We view the VKC-2, like other performance-based tests, as a measure of everyday functional capacity, making it well-suited for between-participant comparisons, staging, and tracking change over time. Questionnaires assess real-world functioning, which is highly unconstrained, with task demands, motivation, economic resources, social support, and other factors varying widely. Thus, in clinical practice, the VKC-2 could be used alongside questionnaires to provide a comprehensive evaluation of everyday function across contexts.

Significant associations between VKC-2 scores and conventional questionnaires of everyday function support the clinical relevance of the VKC-2 measures. Differences between participants with healthy cognition and those with MCI were small to moderate, with absolute differences amounting to only a few seconds on some scores. Such differences may reflect subtle processing difficulties that lead to inefficiency and increased cognitive load, which could accumulate over the course of a day. We acknowledge, however, that direct evidence that the mild cognitive difficulties captured by the VKC-2 translate to meaningful impacts on everyday tasks is currently lacking. Further validation using ecological momentary assessment or digital phenotyping via wearables (or both) would provide more direct evidence of the VKC-2 as a measure of real-world everyday function.

In addition to validity analyses, the reliability of the VKC-2 represents an important novel contribution of this study. To our knowledge, reliability has not been examined for any prior version of the Virtual Kitchen. Retest reliability estimates (ICC) showed that the automated VKC-2 scores—except for distractor interactions, which occurred very infrequently—were highly stable over a 4-6-week period. ICCs were even stronger when participants who reported notable changes in cognitive abilities were excluded. Strong retest reliability is critical for using the VKC-2 to evaluate meaningful change over time and for clinical trial applications. The VKC-2 tasks (breakfast and lunch) also demonstrated strong internal consistency, supporting the coherence of the combined total VKC-2 scores. Furthermore, correlations and PCA indicate that VKC-2 automated scores reflect a single underlying dimension and can be combined into a composite score representing task efficiency.

Several strengths of the study are worth noting. First, the sample size and inclusion of a substantial proportion of participants (106/236, 44.9%) identifying as Black or African American addresses a critical gap in cognitive assessment research and enhances the generalizability of our findings across the US population. Second, the VKC-2’s portability, automated scoring, and standardized administration protocol offer clear advantages over current functional measures and existing regulatory-approved outcome measures for clinical trials, which often require specialized training, lengthy administration times, and access to informants. The VKC-2 does not require Wi-Fi and can be administered on any commercially available, budget-friendly touchscreen computer. Finally, the efficiency of the VKC-2 compared with conventional cognitive test batteries makes it particularly suitable for busy clinical settings where comprehensive neuropsychological assessments are impractical.

Study limitations also warrant consideration. First, although our sample included substantial racial diversity, the predominance of highly educated participants (mean 15.7 years of education) may limit generalizability to populations with lower educational attainment. Additionally, the sample was majority female (156/236, 66.1%), and participants from racial groups other than Black/African American or White or diverse ethnicities were underrepresented. Second, the community-based sample primarily included older adults with healthy cognition, with only 64 of 236 (27.1%) participants meeting criteria for cognitive impairment. The imbalance in subgroup sizes between participants with healthy versus those with impaired cognition limited statistical power for between-group comparisons and AUC/classification analyses. Therefore, additional studies are needed to replicate these findings in samples with larger groups of individuals with MCI or mild dementia. Third, although the virtual task environment is ecologically valid, it may not capture all real-world functional demands, such as physical fatigue, environmental distractions, or competing task requirements. Fourth, the cross-sectional design limits conclusions about the VKC-2’s ability to detect meaningful change over time or predict clinical outcomes (predictive validity). Finally, direct validation against regulatory outcome measures is necessary before the VKC-2 can be considered an alternative end point in clinical trials.

As noted, future research on the VKC-2 should include longitudinal studies to determine the predictive validity of its scores. It will be important to evaluate whether the VKC-2 outperforms conventional measures in identifying individuals at risk for cognitive and functional decline. However, even if the VKC-2 performs comparably to traditional cognitive tests or questionnaires, it offers important advantages, including greater efficiency and independence from the need for a reliable informant. Another important future direction is validation of the VKC-2 against biomarkers of neurodegenerative disease. Holmqvist and colleagues [48] demonstrated strong correlations between VKC-2 scores and magnetic resonance imaging–derived measures of cerebral white matter hyperintensities, a biomarker of small vessel disease associated with brain aging and neurodegeneration. Ongoing studies are examining associations between VKC-2 scores and additional biomarkers, including Alzheimer disease–specific positron emission tomography and blood markers. Finally, automated VKC-2 scores that capture task accomplishment are under development, which will further enhance the utility of the VKC-2 by providing a detailed characterization of everyday task performance patterns [85]. Future implementation research should examine and address potential barriers to VKC-2 adoption, including variability in technology literacy, digital skill levels, and computer-related anxiety among diverse older adults [91], as well as strategies for seamless integration into existing clinical workflows.

In conclusion, there is growing interest in the development of digital assessments of cognition, including digitized versions of traditional cognitive tests, smartphone- and tablet-based cognitive assessments, and VR [92]. Digital, performance-based assessments of everyday tasks, such as the VKC-2, extend this trend to meaningful measures of everyday function. The VKC was designed to address weaknesses of conventional functional measures by providing an objective, standardized, and highly efficient assessment that does not rely on informant reports. The VKC-2 requires approximately 15-20 minutes to administer, is suitable for the full spectrum of cognitive aging—from healthy aging to mild dementia—and includes tasks (breakfast and lunch) that have been extensively studied and shown to be highly familiar to older adults [27,28,32,47,93]. The VKC-2 can be administered on a portable laptop without the need for additional objects or supplies, including a VR headset, avoiding limitations associated with cybersickness and confusion. The touchscreen interface provides a more natural interaction than a mouse or joystick for older adults [94]. Finally, the VKC-2 provides sensitive and detailed performance analysis, including time to completion and measures of performance efficiency derived from the touchscreen, eliminating the need for video recording and human coders. Older adult participants in this study were able to use the touchscreen interface, understood the instructions, did not require extensive training, and performed the tasks consistently with expectations based on data from the Real Kitchen [35]. The VKC-2 shows strong potential as an ecologically valid and scalable tool for capturing everyday functional capabilities in people with healthy cognition, MCI, and mild dementia across various settings, including large longitudinal studies, health clinics, and clinical trials.

## Appendix

Multimedia Appendix 1 Additional analysis.

## Abbreviations

ANCOVA: analysis of covariance
AUC: area under the curve
ECog-12: 12-item Everyday Cognition Scale
FAQ: Functional Activity Questionnaire
IADL-C: Instrumental Activities of Daily Living—Compensation
ICC: intraclass correlation coefficient
IPS: In-Plane Switching
Knight-PACC: Knight-Preclinical Alzheimer Cognitive Composite
MCI: mild cognitive impairment
mKnight-PACC: modified Knight-Preclinical Alzheimer Cognitive Composite
NAT: Naturalistic Action Test
PCA: principal component analysis
QHD+: Quad High Definition Plus
VKC: Virtual Kitchen Challenge
VKC-2: Virtual Kitchen Challenge—Version 2
VR: virtual reality

## Data Availability

The dataset analyzed for this study will be made available in the Virtual Kitchen Challenge Repository. In the interim, data requests may be sent to the corresponding author (TG).
